# Development and application of three-tiered nuclear genetic markers for basal Hexapods using single-stranded conformation polymorphism coupled with targeted DNA sequencing

**DOI:** 10.1186/1471-2156-7-11

**Published:** 2006-02-22

**Authors:** Ryan C Garrick, Paul Sunnucks

**Affiliations:** 1 Department of Genetics, Biological Sciences Building 1, La Trobe University, Plenty Road, Bundoora, VIC 3086, Australia; 2 Australian Centre for Biodiversity Analysis, Policy & Management, School of Biological Sciences, Monash University, Clayton, VIC 3800, Australia

## Abstract

**Background:**

Molecular genetic approaches have much to offer population biology. Despite recent advances, convenient techniques to develop and screen highly-resolving markers can be limiting for some applications and taxa. We describe an improved PCR-based, cloning-free, nuclear marker development procedure, in which single-stranded conformation polymorphism (SSCP) plays a central role. Sequence-variable alleles at putative nuclear loci are simultaneously identified and isolated from diploid tissues. Based on a multiple allele alignment, locus-specific primers are designed in conserved regions, minimizing 'null' alleles. Using two undescribed endemic Australian Collembola as exemplars, we outline a comprehensive approach to generating and validating suites of codominant, sequence-yielding nuclear loci for previously unstudied invertebrates.

**Results:**

Six markers per species were developed without any baseline genetic information. After evaluating the characteristics of each new locus via SSCP pre-screening, population samples were genotyped on the basis of either DNA sequence, restriction site, or insertion/deletion variation, depending on which assay was deemed most appropriate. Polymorphism was generally high (mean of nine alleles per locus), and the markers were capable of resolving population structuring over very fine spatial scales (<100 km). SSCP coupled with targeted DNA sequencing was used to obtain genotypic, genic and genealogical information from six loci (three per species). Phylogeographic analysis identified introns as being most informative.

**Conclusion:**

The comprehensive approach presented here feasibly overcomes technical hurdles of (i) developing suitably polymorphic nuclear loci for non-model organisms, (ii) physically isolating nuclear allele haplotypes from diploid tissues without cloning, and (iii) genotyping population samples on the basis of nuclear DNA sequence variation.

## Background

In non-model invertebrates for which little or no prior genetic information is available, developing suitably polymorphic nuclear markers represents a considerable impediment. Fortunately, a limited number of conserved exon-primed intron-crossing (EPIC) PCR primer pairs for invertebrates are available [1,2], and diverse PCR-based methods for developing anonymous nuclear markers have been proposed (e.g. [3-6]). But perhaps an even greater challenge is developing a suite of genetic markers with sufficient resolution at an appropriate temporal and spatial scale to answer the biological question at hand [7]. Whereas genotype and allele frequencies can change over relatively few generations in diploid sexual species, DNA sequences accumulate mutations over longer timescales. For these reasons, obtaining three hierarchical levels of genetic information (i.e. genotypic, genic and genealogical, *sensu *Sunnucks [7]) from the same marker is highly desirable. But before 'three-tiered' markers can be routinely applied, the methodological hurdle of physically isolating nuclear allele haplotypes one at a time from diploid tissues must be overcome [8-10]. SSCP offers a feasible alternative to costly and labor-intensive cloning of PCR products using a biological vector [11], and when coupled with targeted DNA sequencing, SSCP is an efficient technique for extracting three levels of genetic information simultaneously (e.g. [5,12-15]). Here, we present a comprehensive approach for generating suites of codominant nuclear loci that combines the strengths of diverse PCR-based marker development methods (above), selecting the most appropriate genotyping assay for newly-developed loci prior to large-scale screening, and extracting three levels of genetic information simultaneously.

### Limitations of microsatellites in phylogeographic studies

Understanding what factors influence present-day spatial patterns of genetic diversity, and predicting how a species is likely to respond to environmental change, necessitates an appreciation of processes that have molded its evolutionary history [10]. The use of microsatellites in conjunction with mitochondrial DNA (mtDNA) sequence and frequency data is now commonplace in animal phylogeographic studies. However, isolation of microsatellites from certain invertebrate groups is notoriously difficult (e.g. Onychophora, [16]; Lepidoptera, [17,18]; tenebrionid beetles, [19]), and once obtained, non-amplifying 'null' alleles, size homoplasy, and low levels of polymorphism can severely undermine their utility. For example, after considerable effort, five of 11 microsatellite loci developed by van der Wurff [20] for the Collembolon *Orchesella cincta *were essentially unusable, and three of the remaining six loci had four or fewer alleles from 376 individuals assayed [21]. In addition to low allelic diversity, which may be typical of microsatellite loci in many insects [22], these markers are poorly-suited to inference of genealogical relationships given that the mechanism of mutation affecting repeat regions is often ambiguous, and alleles can be very difficult to sequence.

### Nuclear gene phylogeography

A complete understanding of how historical, demographic and selective processes have molded phylogeographic patterns can only be achieved via comparisons across multiple unlinked loci [8,10,23,24]. Inferences of population history drawn from a single locus are likely to fail to detect some major historical events that shaped the present-day population structure of an organism [24], and accounting for the normal stochastic variance among loci is a major difficulty in phylogeographic studies that do not exploit the signal carried by autosomal genes [23]. To date such datasets have been relatively scarce owing to difficulties obtaining nuclear markers, and then collecting genotypic, genic and genealogical information from the same locus – this is especially true of invertebrate phylogeographic studies (but see [25,26]).

### Log-dwelling 'giant' Collembola – a case study

While there is currently no simple solution to obtaining nuclear markers from non-model animals, the comprehensive strategy presented here worked well with challenging organisms from a barely studied taxon: log-adapted, slime mould-grazing 'giant' Collembola in the family Neanuridae. Based on morphology, dispersal abilities of two as yet undescribed species (Pseudachorutinae n. gen. n. sp. and *Acanthanura *n. sp.) are presumed to be poor given these animals lack a spring organ, are soft-bodied and thus extremely susceptible to desiccation, and have strong preferences for cool, moist microhabitats (personal observation). Molecular genetic techniques may provide the only means for studying aspects of the population biology of these animals given that direct observation, radio-tracking, and capture-mark-recapture are not feasible for small, patchily-distributed log-dwelling invertebrates.

Six codominant, polymorphic nuclear markers per species were developed using a five-step marker development procedure. Briefly, segments of the nuclear genome are amplified from several individuals under low-stringency PCR conditions using a bank of random amplified polymorphic DNA (RAPD), EPIC and microsatellite primers (step 1). The performance of primer pairs is evaluated based on presence/absence of amplified DNA fragments of corresponding sizes from all individuals assayed, and 'target' bands identified (step 2). Additional DNA templates are added to the subset and, where possible, higher stringency PCR conditions used to preferentially amplify target bands (step 3). Multiple representatives of the target product are excised from the gel and used as template for isotope incorporation PCR (step 4). Sequence- and/or size-variable alleles are simultaneously identified and isolated using SSCP, reamplified from gel slices, and sequenced (step 5). Based on a multiple allele alignment, locus-specific primer pairs are designed in highly conserved regions. SSCP coupled with targeted DNA sequencing was used to obtain genotypic, genic and genealogical information from six loci, for moderate to large population-genetic sample sizes (102 to 378 individuals). The method yielded a robust dataset well suited to emerging and traditional population-genetic and phylogeographic analyses. To illustrate the interplay between molecular population biology approaches and the utility and limitations of the three levels of genetic information, we employed a subset of exploratory analyses commonly used in molecular ecology and phylogeography.

## Results

### Identity and function of nuclear gene regions

Twelve species-specific primer pairs that reliably amplify nuclear loci without appreciable null allele frequencies were developed using SSCP coupled with targeted DNA sequencing, in conjunction with four PCR-based marker development procedures (methods A-D, see Methods; Table 1). The gene regions amplified are unlinked and include introns from the conserved nuclear genes *Elongation factor-1α *(*EF-1α*) and *Adenine Nucleotide Transporter *(*ANT*), a member of the *Wingless *(*Wnt*) gene family, and eight anonymous loci. The *Acanthanura *n. sp. *Wnt *locus (*UcWnt*) was presumed protein-coding because alleles displayed open reading frames (ORFs), all observed substitutions were synonymous, and the sole amino acid sequence showed high similarity with *Wnt *orthologues reported for other Arthropods [27]. Since our restriction fragment length polymorphism (RFLP) assay resolved only silent DNA polymorphisms, this marker was considered selectively neutral. All other nuclear loci appeared non-coding. For example, when DNA sequences from several alleles at each locus were translated, stop codons were observed in each reading frame. Further, at size-variable loci, insertion/deletion mutations (indels) were not consistently comprised of contiguous alignment gaps in multiples of three, which would cause frame shifts if translated.

**Table 1:** Six new nuclear loci for each of two Collembola species. Markers were developed using four PCR-based methods (labeled A-D) in conjunction with SSCP plus DNA sequencing. Pseud., Pseudachorutinae n. sp.; *Acanth*., *Acanthanura *n. sp.

Taxon/locus	Primer	Sequence (5' to 3')	Gene amplified	Method	Source of primers used for initial amplification (primer names)
Pseud.					
*Sm2*	Sm2-F^a^	GAAACGGGTGCTGGTTSRAGG	Anon. nDNA	A	Operon Technologies (A07, A09)
	Sm2-R	GGGTAACGCCRTTGGAAACAG			
*Sm4*	Sm4-F	GAATTGGTGGGAGATCTCTC	Anon. nDNA	C	[2] (ATPSα f1, ATPSα r1)
	Sm4-R	TGTCGTCCGTCTATGATTCG			
*Sm6*	Sm6-F	CTGAATGCCGTCGAAACGTAAAC	Anon. nDNA	B	[57] (M49-F), [58] (myz3-R)
	Sm6-R	GTTGGTTTACCTGTTTTAAATG			
*Sm8*	Sm8-F	AGTGGGATTTTAGGATGGCAGG	Anon. nDNA	A/B	[57] (M49-F), Operon Technologies (A07)
	Sm8-R	CCAAGACTAAGATTGAGAAGAAGTC			
*SmEF-1α*	UcEF-F^a^	see below	*EF-1α *intron	C	This study (UcEF-F), [1] (EF2)
	SmEF-R	CCAATMCCACCAATTTTGTAGAC			
*Sm150*	Sm150-F	ATCCTACTCAAAACTCAAG	Anon. nDNA	D	Not applicable
	Sm150-R	ACCCTTGGATTTGGAATC			

*Acanth*.					
*Uc3*	Uc3-F^a^	CAGCGCGGTTTGGGTGTATA	Anon. nDNA	A	Operon Technologies (A07, A10)
	Uc3-R	GTGATCGCAGAAATCCCCGCA			
*Uc180*	Uc180-F^a^	CCAACTCAAGTTCGGATGAC	Anon. nDNA	D	Not applicable
	Uc180-R	CAAAGCGCTTAACTGGGTC			
*Uc44*	Uc44-F	GATTATTACCAATCGCTATTGG	Anon. nDNA	A	Operon Technologies (A07, A09)
	Uc44-R	GGGTAACGCCAATTTAAGGTG			
*UcEF-1α*	UcEF-F^a^	CCGAGAAGATGTCCTGGTTC	*EF-1α *intron	C	[1] (EF0, EF2)
	UcEF-R	CGGGCACTGTTCCAATCC			
*UcANT*	UcANT-F	CAGTGTCTCMGTKCAAGGAATC	*ANT *intron	C	[2] (ANTf1, ANTr1)
	UcANT-R	MTTGGGCGATWAKCCAKGAAAC			
*UcWnt*	UcWnt-F	AGAATAAGTTCCGTCGTGCTG	*Wnt*	C	[59] (wg1a, wg2a)
	UcWnt-R	GGATTGGAGTCGCAGTAGGTC			

^a^Primer used for sequencing.

### Evaluation of marker development and genotyping methods

The comprehensive approach solved a number of commonly-encountered technical difficulties. For example, unique alleles were conveniently identified and isolated for sequencing without cloning, multiple nuclear loci suitable for constructing allele phylogenies were obtained with gametic phase of segregating sites recovered with certainty (c.f. haplotype-subtraction, [28]), and three levels of genetic information were simultaneously extracted from the same marker.

For each polymorphic marker obtained using the approach described in Methods, an average of 2.3 PCR product-generating primer pairs were trialed, 13.3 DNA sequences evaluated, and 2.2 locus-specific primer pairs designed. Method C was only marginally more successful than method A in producing nuclear loci informative at the population-level. Generally, these two methods produced a moderate to large number of 'target' bands in the initial stage of marker development, but a relatively high proportion of these putative alleles were not homologous (Table 2). Accordingly, the two methods that produced the greatest number of loci also had the highest drop-out rate. The converse was true for methods B and D, which use long non-degenerate PCR primer pairs for initial amplification of nuclear DNA (see Methods). Nonetheless, all four methods generated at least two useable markers (Table 1). In our experience, a multifaceted approach to marker development seems to be the most efficient strategy, and this philosophy underpins the general-purpose five-step procedure presented here.

**Table 2:** Attributes of marker development methods, genotyping assays, and DNA sequence-yielding nuclear loci. Qualitative assessment of relative success scored using the following three categories: +++, high/many; ++ moderate; + low/few. Genotyping assays are: SSCP, single-stranded conformation polymorphism coupled with targeted DNA sequencing; RFLP, restriction fragment length polymorphism; Indel, insertion-deletion mutation detection.

Development	No. target bands generated (step 2)	Putative allele homology (step 5)	No. loci produced
Method A	+++	+	++
Method B	+	+++	+
Method C	++	++	+++
Method D	+	+++	+

Assay	Within-population variability	Commitment	
		
		Cost	Time

SSCP	+++	+++	+++
RFLP	+	+	++
Indel	++	++	+

Locus type	Within-population variability	Sequence divergence	Phylogeographic signal

*EF-1α *introns	+++	+	+++
Anon. nDNA	++	+++	++

Genotyping methods selected for each locus based on SSCP pre-screening are given in Table 3. In general, the procedure of resolving indel variation only (as commonly applied in molecular ecology) was seldom selected, since it would fail to detect considerable genetic variation. Owing to the unexpectedly large number of substitutions within short (<300-bp) fragments, the number of alleles per locus resolved by SSCP coupled with targeted DNA sequencing was between three and 10 times greater than that which could be resolved from indel variation alone. RFLP generally resolved the fewest alleles (Table 3), only partly due to constraints posed by the prohibitive cost of some enzymes. Levels of within-population variation mirrored trends evident from assessment of total number of alleles detected by each of the three genotyping assays. Genetic resolution was roughly proportional to cost and time commitment, although where possible, genotyping population samples on the basis of indel variation may be more efficient than RFLP assays (Table 2).

**Table 3:** Assays selected for genotyping population samples using newly-developed nuclear loci, following SSCP pre-screening. Taxon and genotyping assay abbreviations follow Table 1 and Table 2, respectively. The genetic basis of polymorphism at each locus was determined via sequencing. The *Sm8 *locus was initially assayed using SSCP [31], but here we deemed RFLP more appropriate. The latter dataset was used for Bayesian clustering, calculating allelic richness, and estimating phenetic relationships among populations.

Taxon/locus	Fragment length (bp)^a^	Substitutions	Indels^b^	Total no. of mutations	Genotyping assay	No. of alleles resolved	No. of animals screened	GenBank accession no.
Pseud.								
*Sm2*	161–189	17	4	21	SSCP (*HhaI*)	13	378	DQ322462-DQ322474
*Sm8*	232–234	21	4	25	SSCP/RFLP (*HinfI*, *NlaIV*)	12/4	102/377	DQ322492-DQ322503
*SmEF-1α*	228–232	20	5	25	SSCP	21	373	DQ322508-DQ322528
*Sm6*	61–76		6		Indel	5	380	DQ322485-DQ322491
*Sm4*	231–325		10–12		Indel	4	379	DQ322475-DQ322484
*Sm150*	129–131		2		Indel	2	380	DQ322504-DQ322507

*Acanth*.								
*Uc180*	92–96	10	2	12	SSCP	9	201	DQ322552-DQ322560
*Uc3*	146–150	24	2	25	SSCP	17	203	DQ322529-DQ322545
*UcEF-1α*	264–266	26	1	27	SSCP (*HincII*)	21	203	DQ322566-DQ322586
*Uc44*	117–133		5		Indel	6	203	DQ322546-DQ322551
*UcANT*	173–174				RFLP (*AluI*, *DraI*)	3	203	DQ322561-DQ322565
*UcWnt*	139				RFLP (*FokI*, *NlaIII*)	3	203	DQ322587-DQ322590

^a^Allele size range minus primers and ambiguous sequence.

^b^Contiguous aligment gaps were treated as a single indel, *Sm4 *alleles cannot be unambiguously aligned. For loci assayed using the indel genotyping procedure, the number of indels observed in a subset of representative allele sequences in given.

### Identification of population structure and assignment of individuals

**Figure 1:**
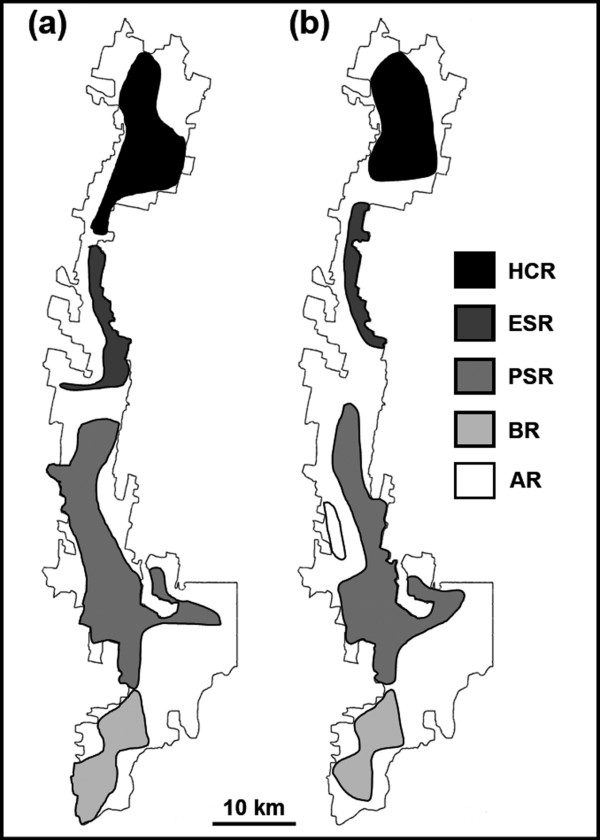
**Spatial distribution of Collembola populations**. (a) Pseudachorutinae n. sp., (b) *Acanthanura *n. sp. Populations were identified *a posteriori *on the basis of six-locus genotype clustering (supplemented by phylogenetic analysis of mtDNA). HCR, Harolds Cross Region; ESR, Eastern Slopes Region; AR, Anembo Region; PSR, Pikes Saddle Region; BR, Badja Region. For a description of physical landscape features and the likely palaeoclimatic history of Tallaganda, see Garrick *et al*. [31].

Bayesian clustering analysis of the two six-locus nuclear datasets (supplemented by phylogenetic analysis of mtDNA) identified four populations of Pseudachorutinae n. sp., and five populations of *Acanthanura *n. sp. at Tallaganda (Figure 1). For both species, most individuals were strongly assigned to a single genetic population (sample sizes given in Table 4), admixed individuals tended to occur at or near contact zones, and putative migrants were rare.

**Table 4:** Population allele frequencies. Taxon and population abbreviations follow Table 1 and Figure 1, respectively. Allele frequencies for the *Sm8 *locus are based on the RFLP genotyping assay.

Taxon/locus		Population				Taxon/locus		Population				
					
	Allele	HCR	ESR	PSR	BR		Allele	HCR	ESR	AR	PSR	BR
	
Pseud.						*Acanth.*						
*Sm2*	*N*	*68*	*79*	*106*	*61*	* Uc3*	*N*	*24*	*19*	*15*	*56*	*48*
	A1	0.54	0.99	---	---		A1	---	---	---	0.02	---
	A2	0.08	---	0.39	---		A2	0.19	---	0.03	---	---
	A3	---	---	0.61	---		A4	---	---	0.23	---	---
	A4	---	---	---	0.90		A5	---	---	0.03	---	---
	A5	0.35	---	---	---		A6	0.81	1.00	0.27	0.76	0.52
	A8	---	---	---	0.02		A7	---	---	---	0.03	---
	A9	---	---	---	0.05		A8	---	---	0.10	0.04	---
	A10	---	---	---	0.02		A9	---	---	---	0.15	---
	A11	---	---	---	0.02		A10	---	---	---	0.01	0.01
	A12	0.03	---	---	---		A11	---	---	---	---	0.04
	A13	---	0.01	---	---		A12	---	---	---	---	0.28
							A13	---	---	0.07	---	0.12
*Sm8*	*N*	*68*	*79*	*106*	*61*		A14	---	---	---	---	0.03
	A1	0.15	0.48	0.72	0.01		A15	---	---	0.13	---	---
	A2	0.85	0.02	0.28	---		A16	---	---	0.07	---	---
	A3	---	0.50	---	0.88		A17	---	---	0.07	---	---
	A4	---	---	---	0.12							
						*Uc180*	*N*	*22*	*19*	*15*	*56*	*48*
*SmEF-1α*	*N*	*68*	*78*	*106*	*61*		A1	---	---	1.00	---	---
	A1	0.07	---	0.07	---		A2	---	---	---	0.01	---
	A2	0.07	---	---	---		A3	---	---	---	---	0.01
	A3	---	---	0.05	---		A4	0.50	---	---	---	---
	A5	---	0.07	---	---		A5	0.18	---	---	---	---
	A6	0.85	0.89	---	---		A6	---	0.97	---	---	---
	A7	---	---	0.09	---		A7	---	0.03	---	---	---
	A8	---	0.05	0.34	---		A8	0.21	---	---	0.99	0.99
	A10	---	---	---	0.37		A9	0.11	---	---	---	center---
	A11	---	---	---	0.06							
	A12	---	---	---	0.03	*UcEF-1α*	*N*	*24*	*19*	*15*	*56*	*48*
	A13	---	---	---	0.02		A1	---	0.03	---	0.59	---
	A14	---	---	---	0.01		A2	0.21	---	---	---	---
	A15	---	---	0.04	---		A3	0.52	0.92	---	---	---
	A16	---	---	---	0.17		A4	0.02	0.03	---	---	---
	A17	---	---	---	0.02		A5	---	---	1.00	---	---
	A18	---	---	0.41	---		A6	0.15	---	---	---	---
	A19	---	---	---	0.33		A7	---	---	---	0.16	---
	A20	0.01	---	---	---		A8	---	---	---	0.02	---
	A21	---	---	---	0.01		A9	---	---	---	0.13	0.06
							A10	---	---	---	0.01	---
*Sm6*	*N*	*68*	*79*	*106*	*61*		A11	---	---	---	0.01	---
	A1	1.00	0.60	0.35	---		A12	---	---	---	---	0.81
	A2	---	---	---	0.07		A13	---	---	---	0.08	---
	A3	---	0.41	0.04	---		A14	---	---	---	---	0.09
	A4	---	---	0.61	0.84		A15	0.02	---	---	---	---
	A5	---	---	---	0.09		A17	---	---	---	---	0.02
							A19	---	0.03	---	---	---
*Sm4*	*N*	*68*	*79*	*106*	*60*		A20	---	---	---	---	0.01
	A1	---	0.35	---	0.08		A21	0.08	---	---	---	---
	A2	---	0.21	0.09	---							
	A3	1.00	0.44	0.91	0.88	*UcANT*	*N*	*24*	*19*	*15*	*56*	*48*
	A4	---	---	---	0.03		A1	1.00	1.00	---	0.55	---
							A2	---	---	---	0.45	1.00
*Sm150*	*N*	*68*	*79*	*106*	*61*		A3	---	---	1.00	---	---
	A1	1.00	1.00	0.08	1.00							
	A2	---	---	0.93	---	*UcWnt*	*N*	*24*	*19*	*15*	*56*	*48*
							A1	1.00	1.00	1.00	0.24	0.27
							A2	---	---	---	0.18	0.03
							A3	---	---	---	0.58	0.70
						
						*Uc44*	*N*	*24*	*19*	*15*	*56*	*48*
							A1	0.71	0.32	0.03	---	---
							A2	---	0.03	0.57	---	0.01
							A3	0.08	0.37	---	---	---
							A4	---	---	---	0.01	---
							A5	0.21	0.29	0.40	0.99	0.97
							A6	---	---	---	---	0.02

### Levels of polymorphism and phenetic relationships among populations

The new nuclear markers displayed a moderate to large number of alleles (Table 4), and were informative over fine spatial scales (Figure 1). Allelic richness varied considerably among populations and loci. In both species, loci assayed via SSCP coupled with targeted DNA sequencing generally yielded the highest mean within-population allelic richness values, while the converse was true for loci assayed using RFLP. With the exception of *Sm150*, markers assayed for indel variation provided an intermediate level of resolution (Table 5).

**Table 5:** Within- and among-population genetic variation. Allelic richness based on a standardized sample size of 14 diploid individuals. Taxon and population abbreviations follow Table 1 and Figure 1, respectively.

Taxon/population	Locus/allelic richness (A)						Percentage loci polymorphic (*P*)
Pseud.	*SmEF-1α*	*Sm2*	*Sm8*	*Sm4*	*Sm6*	*Sm150*	
HCR	3.02	3.54	2.00	1.00	1.00	1.00	50
ESR	2.65	1.18	2.45	3.00	2.00	1.00	83
PSR	5.29	2.00	2.00	1.95	2.73	1.91	100
BR	5.67	3.02	2.21	2.60	2.84	1.00	83
Mean	4.16	2.43	2.16	2.14	2.14	1.23	

*Acanth*.	*UcEF-1α*	*Uc3*	*Uc180*	*UcANT*	*UcWnt*	*Uc44*	
HCR	5.14	2.00	4.00	1.00	1.00	2.98	67
ESR	3.21	1.00	1.74	1.00	1.00	3.74	50
AR	1.00	8.86	1.00	1.00	1.00	2.93	33
PSR	4.86	3.96	1.25	2.00	3.00	1.25	100
BR	3.64	4.68	1.29	1.00	2.65	1.79	83
Mean	3.57	4.10	1.85	1.20	1.73	2.54	

**Figure 2:**
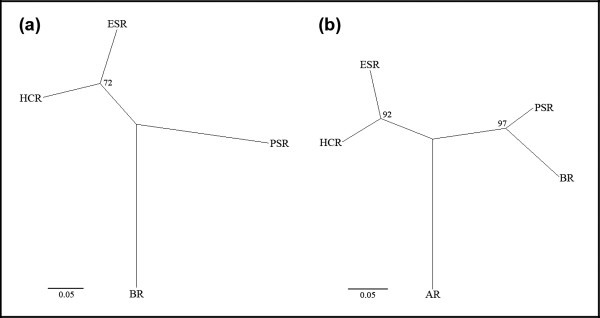
**Phenetic relationships among Collembola populations**. (a) Pseudachorutinae n. sp., (b) *Acanthanura *n. sp. Genetic distance was measured using *D*_*c*_, calculated from population allele frequencies at six nuclear loci. Numbers at nodes of the NJ phenogram indicate bootstrap support.

Neighbor-Joining phenograms showed that for both species of Collembola, geographically proximate populations group together (Figure 2). Under an infinite sites model, Cavalli-Sforza & Edwards' [29] chord distance (*D*_*c*_) increases approximately linearly with time when population divergences are low (*D*_*c *_~0.2 – 0.4, [30]). In the present study, the range of pair-wise *D*_*c *_values was 0.12 to 0.41, so we can tentatively propose evolutionary scenarios relating to the temporal sequence of divergence events. For example, phenetic relationships among Pseudachorutinae n. sp. populations could indicate that BR was separated from the rest at the deepest split, closely followed by PSR, whereas HCR/ESR recently diverged. Similarly, the AR may have been the most ancient divergence among extant *Acanthanura *n. sp. populations, while separations between HCR/ESR, and between PSR/BR, are more recent events.

### Tests of recombination and selective neutrality

At *SmEF-1α*, significant evidence of recombination (*P *< 0.01, Table 6) was detected for three pair-wise comparisons involving four of the 21 alleles resolved (A13–A16, A13–A18 and A3–A16). Because three of these alleles occur only in the BR population (i.e. A13, A16 and A18, Table 4), which is likely to have been isolated for a long period of time [31], phylogeographic signal may be relatively unaffected [25]. Therefore only A3 was excluded from phylogenetic analysis of *SmEF-1α*. All other loci assayed via SSCP coupled with targeted DNA sequencing were free of detectable recombination (Table 6).

**Table 6:** Tests of recombination and selection. Tajima's *D *[30] *P*-values are *P < 0.05, **P < 0.01, and ns, not significant at the 0.05 level; NP, no polymorphism. Taxon and population abbreviations follow Table 1 and Figure 1, respectively.

Taxon/locus	Recombination		Tajima's *D*					
		
	Inner frag. *P*-value	Outer frag. *P*-value	HCR	ESR	AR	PSR	BR	All populations
Pseud.								
*Sm2*	0.28	1.00	1.65 ns	-0.98 ns		1.83 ns	-2.14*	1.22 ns
*Sm8*	0.36	0.51	2.86**	0.34 ns		1.09 ns	-0.60 ns	0.49 ns
*SmEF-1α*	<0.01	1.00	-0.48 ns	-0.92 ns		0.77 ns	-0.36 ns	-0.22 ns

*Acanth*.								
*Uc180*	0.56	0.78	0.33 ns	-1.49 ns	NP	-1.02 ns	-1.03 ns	-0.99 ns
*Uc3*	0.36	0.08	0.52 ns	NP	2.46*	-1.24 ns	-0.84 ns	-1.63 ns
*UcEF-1α*	0.89	1.00	-0.21 ns	-2.18**	NP	0.18 ns	-0.48 ns	-0.46 ns

Using the criteria for inferring non-neutrality of loci outlined in Methods, Tajima's *D *did not provide strong evidence for selection acting on any of the six DNA sequence markers. Although there were two instances of significantly negative values of *D *(often associated with selection against rare deleterious alleles), observations from other loci show that this is also consistent with population demographic changes (Table 6). Two cases of significantly positive values of *D *were evident. While we cannot discount population-specific balancing or diversifying selection, this result may also be an artifact of non-random sampling (e.g. family structure within samples collected from a single rotting log). Accordingly, we considered these loci largely unaffected recombination and selection, at least at the among-population level.

### Phylogeographic analysis

**Figure 3:**
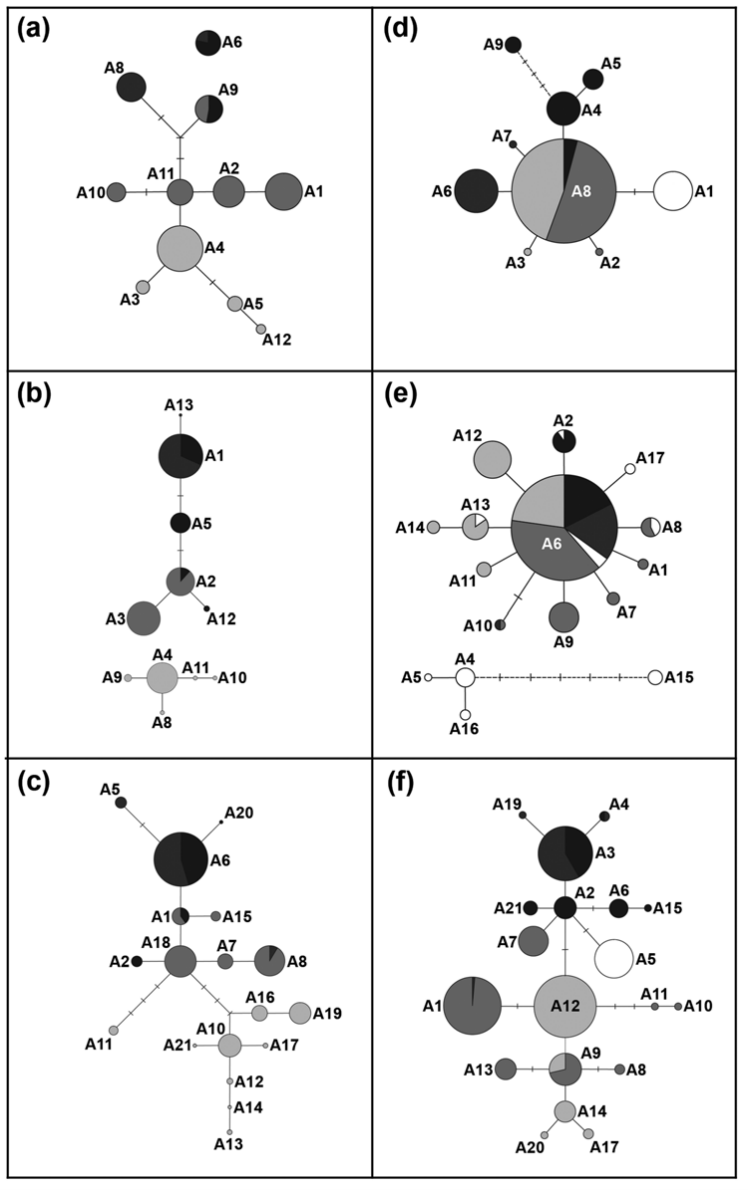
**Phylogenetic relationships among alleles estimated using statistical parsimony (SP)**. Pseudachorutinae n sp. (a) *Sm8*, (b) *Sm2*, (c) *SmEF-1α*; *Acanthanura *n. sp. (d) *Uc180*, (e) *Uc3*, (f) *UcEF-1α*. For each SP cladogram, circles represent unique alleles (labeled following Table 4). The size of each circle is proportional to the frequency of that allele across all populations. Relative frequencies of alleles in each population are indicated by pie charts (color-coded following Figure 1). Single black lines represent one mutational step, short crossbars represent inferred alleles that were not sampled or are extinct. Dashed lines identify connections that failed to satisfy the 95% confidence criterion by a single mutational step.

Comparison of gene tree topologies within species showed common patterns of deep molecular divergences among (at least some) populations, and high spatial localization of most alleles (Figure 3). A qualitative assessment of the degree of phylogeographic signal produced by each of the six nuclear loci revealed that, for both Collembolons, *EF-1α *introns were the most informative (Figure 3, Table 2). Although these two loci did have the most alleles, they also had the lowest maximum sequence divergences among alleles relative to anonymous nuclear loci (*SmEF-1α *= 3.6% uncorrected *p *c.f. *Sm2 *= 5.9% and *Sm8 *= 5.5%; *UcEF-1α *= 3.0% c.f. *Uc180 *= 5.4% and *Uc3 *= 10.7%). As a consequence, all alleles at intron loci were parsimoniously connected at 95% confidence to form a single cladogram. This is a desirable property when estimating the root of an intraspecific gene tree [32], which is a critical aspect of nested clade analysis (NCA, [33]).

For the three Pseudachorutinae n. sp. nuclear sequence markers assayed, BR alleles tended to be quite divergent from those found in other populations (Figure 3a–c). This apparent deep divergence is consistent with population relationships estimated from genic data (Figure 2), and mtDNA data [31]. *Acanthanura *n. sp. sequence markers showed that among-locus variability can be considerable (Figure 3d–f). For example, although *Uc180 *and *Uc3 *allele phylogenies are topologically similar (i.e. star-shaped), the former locus shows a monophyletic clade exclusive to HCR and two populations are fixed for a single unique allele, while the latter locus has a monophyletic clade confined to AR, and no population is fixed for a unique allele. However, the greatest contrast is between *UcEF-1α*, which has three major hubs in the cladogram, each with relatively narrow spatial distributions, and the two anonymous nuclear loci, which both have a single high-frequency and geographically widespread allele at the center of those networks.

## Discussion

The primary methodological advance provided by the approach presented here lies in the ability to develop nuclear markers with specific characteristics tailored to the research question at hand, identify genotyping assays that achieve a balance between efficiency and resolving power prior to large-scale screening, and obtaining three levels of genetic information simultaneously from the same marker. A secondary advance comes from combining the strengths of diverse PCR-based marker development protocols (Background) in a simple five-step procedure that does not require cloning.

Using this approach, we obtained a suite of polymorphic nuclear markers for two undescribed Collembola relatively quickly and cheaply. These markers were informative over fine spatial scales, appeared to be non-coding (with the exception of *UcWnt *– see Results), were free of appreciable null allele frequencies, and as far as we can determine without pedigreed material, alleles were segregating in a Mendelian fashion. Together with targeted DNA sequencing, SSCP was an effective technique for obtaining genotypic, genic and genealogical information from six nuclear genes for moderate to large population-genetic sample sizes. These loci showed no consistent significant departure from neutrality over all populations, were mostly free of detectable recombination, and were therefore suitable for constructing nuclear gene trees using standard phylogenetic procedures. Indeed, the datasets obtained here are well-suited to variety of emerging coalescent-based statistical phylogeographic analyses [34-36], and NCA.

### The role of SSCP in development and application of three-tiered nuclear markers

Single-stranded conformation polymorphism was effective for physically isolating nuclear allele haplotypes from diploid tissues. While cloning of PCR products also provides a means of separating size-invariable alleles, this procedure under many jurisdictions requires a dedicated Physical Containment level 1 laboratory and formal permission from gene technology regulators, is labor-intensive, and expensive (e.g. requires cloning vectors and competent bacteria). Further, to achieve a moderate level of certainty regarding genotype assignment, at least four clones per PCR product are usually sequenced [26], but the number required to detect weakly amplifying alleles may be considerably greater, and thus prohibitively expensive [9]. An additional drawback is that cloned sequences can have a greater propensity to reveal artifacts such as *Taq *DNA polymerase error and PCR recombinants [37].

The SSCP procedure provides researchers with the advantage of being able to identify putative sequence-variable alleles directly from autoradiograph phenotypes. Thus, only strategically-selected bands need to be sequenced. Further, given the high sensitivity of radioisotope-based methods for viewing low-concentration DNA [10], weakly amplifying alleles can be detected with greater efficiency than with cloning (for laboratories without access to radioisotope, silver-staining should produce comparable SSCP results, and also permits bands to be excised and reamplified in PCR [15]). Similarly, SSCP pre-screening provides valuable information on levels of polymorphism at a newly-developed locus, enabling informed decisions about its suitability for addressing the research question, and for identifying cost- and time-efficient genotyping assays.

While we found SSCP to be a valuable technique for genotyping population samples on the basis of DNA sequence variation at multiple nuclear loci, we must introduce a note of caution. When there is a large number of individuals (*c*. >300) to be assayed, and/or a large number of alleles (*c*. >15) at a locus, a substantial time commitment may be required. The cost of sequencing can increase considerably when there are many unique gel phenotypes (putative genotypes) present on each autoradiograph, or single-stranded DNA (ssDNA) adopts multiple conformations. Running individuals that are likely to share alleles on the same gel alleviates the former problem, while the latter situation seems to arise mostly when primers contain degenerate nucleotide positions, and where possible, we recommend avoiding their use for large-scale screening. Finally, there may be rare occasions where closely-related alleles produce indistinguishable SSCP gel phenotypes. In these cases we have used a RFLP assay to distinguish known alleles (Table 3). In the absence of a diagnostic restriction site, it may be necessary to design primers that amplify alleles singularly.

### Identifying contemporary spatial-genetic patterns and estimating population relationships

Joint analysis of genotypic data revealed population substructuring over fine spatial scales in both Collembola species, consistent with expectations for ecologically-specialized, low-mobility animals. Although genotypic data from multiple loci proved useful for describing contemporary spatial-genetic patterns, it offered little information on the degree of divergence among populations. Having first objectively defined populations *a posteriori *in a spatially explicit manner, we were then able to quantify levels of within-population genetic diversity and estimate population relationships using genic data.

Kalinowski [38] proposed that the total number of independent alleles (i.e. number of alleles at a locus minus one, summed across loci) is a good indicator of precision in genetic distance estimates. Accordingly, loci with many alleles are more efficient in producing good estimates. Nuclear markers developed using the approaches presented here and genotyped using SSCP coupled with targeted DNA sequencing were highly polymorphic (mean 15.5 alleles per locus, Table 3), and so are well-suited to genetic distance-based analyses. In both species, geographically proximate populations tended to be genetically more similar to one another (c.f. distant populations), and population divergences seem to have occurred on different timescales throughout the evolutionary history of these animals at Tallaganda. However, when genetic distances between populations are small, either recent divergence with zero gene flow (isolation model), or ancient divergence with low ongoing gene flow (migration model) can represent equally plausible scenarios [39]. While genic data are useful for quantifying levels of genetic diversity and estimating population relationships, they are unable to provide a full picture that integrates both separation time and gene flow [36]. This can be addressed by analysis of DNA sequence data.

### Nuclear gene phylogeography

Because models of isolation versus migration can lead to similar gene tree topologies [36], the present datasets will be analyzed using likelihood methods, and results presented elsewhere. Nonetheless, phylogenetic analyses demonstrated that all of the new Collembola nuclear loci assayed using SSCP coupled with targeted DNA sequencing contain phylogeographic signal that can be exploited. Interestingly, in both species, the *EF-1α *locus – the only one known to be an intron of a functional gene – had the lowest range of sequence divergences yet the strongest phylogeographic signal (Figure 3, Table 2), possibly indicating purifying selection not detected by Tajima's *D *neutrality tests (Table 6). Indeed, intron polymorphisms are increasingly being recognized as important in gene regulation [40], and it has recently been postulated that the majority of intronic DNA in the genome is likely to be evolving under considerable selective constraint [41].

Although introns offered the greatest phylogeographic resolution for both Collembolons, the value of anonymous nuclear sequence-markers should not be understated. Because different loci can vary widely in their histories [23,24], sampling additional individuals (after a certain point) is far less informative than sampling additional unlinked loci [34,36]. In the present study, comparative analysis of genes within species confirmed that stochastic variance among loci can be considerable. Although there were some notable differences in spatial structuring, *Acanthanura *n. sp. loci *Uc180 *and *Uc3 *both produced star-shaped phylogenies, consistent with expectations for an exponentially growing population [42]. In contrast, *UcEF-1α *showed marked phylogeographic structuring, with multiple putative 'ancestral' allele haplotypes occupying central positions in the cladogram, each associated with a series of closely-related descendants. This case study illustrates that multiple nuclear sequence markers have the potential to radically alter population inferences.

## Conclusion

Recovering intraspecific nuclear gene trees that have not been severely compromised by recombination or selection presents considerable theoretical, biological, and analytical challenges [8-10]. Using the comprehensive SSCP-based approach presented here, we have shown that technical hurdles such as (i) developing suitably polymorphic nuclear loci for non-model organisms, (ii) physically isolating nuclear allele haplotypes from diploid tissues and (iii) genotyping population samples on the basis of DNA sequence variation can be feasibly overcome. While it is true that in some circumstances SSCP coupled with targeted DNA sequencing can become a relatively time-consuming and expensive genotyping procedure (see above), this is probably true of any technique currently available.

## Methods

### Taxon sampling, study site and DNA isolation

The two focal Collembola represent new species, and have been clearly characterized (P. Greenslade, taxonomic descriptions in preparation). No genetic information for animals of the same genera (and in one case, subfamily) was available prior to the present research program. Between 1997 and 2004, 203 specimens of *Acanthanura *n. sp. and 380 Pseudachorutinae n. sp. were collected from 130 rotting logs along a ~100 km transect traversing Tallaganda State Forest (SF), Tallaganda National Park (NP), Deua NP and Badja SF (collectively referred to as 'Tallaganda'), in south-east New South Wales (NSW), Australia. Genomic DNA was isolated using a simplified 2× cetyltrimethylammonium bromide (CTAB) protocol, modified after Murray & Thompson [43].

### Marker development procedures

We combined several previously-published protocols [4-6], modified to eliminate the need for cloning. We divided methods used here into four categories (labeled A-D), and describe a general SSCP-based approach for nuclear marker development.

#### Step 1

Nuclear DNA was amplified via PCR from four conspecific individuals collected from different localities using either (A) randomly selected pairs of short (10-mer) RAPD primers, (B) randomly selected pairs of long (*c*. 20-mer) aphid microsatellite primers, (C) degenerate EPIC primer pairs, or (D) primers designed from anonymous DNA sequences from a Pseudachorutinae n. sp. genomic library (Table 1). All reactions were performed using a Corbett Palmcycler CGI-960 in 10 μL volumes containing 16 μM ammonium sulphate, 68 mM Tris-HCl (pH 8), 10 mM β-mercaptoethanol, 5% bovine serum albumin, 10 mg/mL (Progen), 2 mM magnesium chloride, 200 μM each dNTP, 0.5 μM each primer for two-primer reactions, or 1.0 μM for single-primer reactions (see below), 0.5 units of *Taq *DNA Polymerase (Promega), and 1 μL template DNA. PCR profiles were: (A) 94°C 30 s, 37°C 30 s, 72°C 30 s (35 cycles); (B) 94°C 3 min (1 cycle), 94°C 30 s, 52°C 1 min, 68°C 8 min (35 cycles), 68°C 8 min (1 cycle); (C) and (D) 94°C 2 min (1 cycle); 94°C 30 s, 40 – 58°C 30 s, 72°C 1 min (35 cycles), 72°C 2 min (1 cycle). For methods C and D, we used an annealing temperature gradient: each template was amplified using 40, 46, 52 and 58°C.

#### Step 2

To evaluate primer pairs and identify 'target' bands, PCR products were electrophoresed through 6% non-denaturing acrylamide gels (8 cm) at 200 V for 30 min, stained with ethidium bromide, and viewed using ultraviolet light. We used a 100-bp ladder (Fermentas) to estimate fragment sizes. For methods A and B, one of the four templates was also used in reaction mixtures that contained each primer alone. Bands that amplified only in two-primer reactions have different primers on each end, and can be sequenced [4]. Bands of approximately the same size (± 50-bp) that amplified from all four templates, and were >100-bp were considered for further development (smaller fragments are unsuitable for designing internal locus-specific primers).

#### Step 3

DNA template from four individuals was added to the subset. For methods A and B, PCRs were repeated as in step 1. For methods C and D, the two consecutive annealing temperatures that gave good amplification of 'target' bands in step 2 defined upper and lower limits of a second gradient profile, again using four annealing temperatures per template, but now increasing in 2°C increments (c.f. 6°C, step 1). All other PCR conditions followed step 1.

#### Step 4

PCR products were electrophoresed and viewed as in step 2. Target bands were excised using a scalpel, gel slices were soaked in 20 μL 1× TE (10 mM TrisHCl pH 8.0, 1 mM EDTA pH 8.0), microfuged at 13,000 *g *for 30 s, and incubated at 45°C for 30 min. From each gel slice, 1 μL of the supernatant was used as template for reamplification with isotope incorporation, where 0.05 μL [α^33^P]-dATP (10 mCi/mL) was added to each 10 μL reaction mixture (step 1). We employed the same PCR profile in step 1, using the lowest annealing temperature identified in step 3 where applicable.

#### Step 5

**Figure 4:**
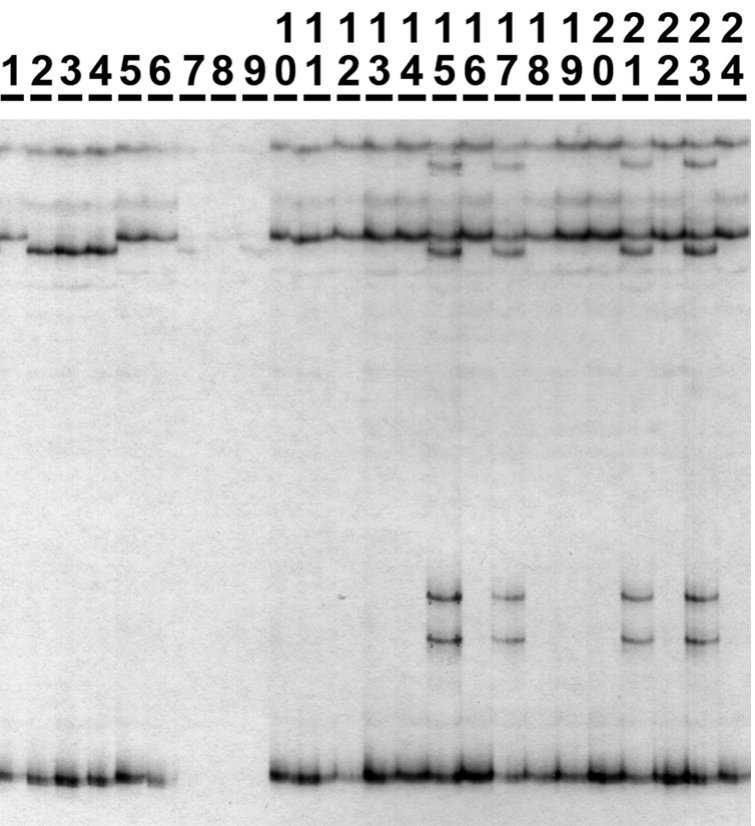
**Autoradiograph showing some of the SSCP gel phenotypes observed at the *Uc3 *locus**. Three distinct patterns are evident, and sequencing confirmed that these correspond with three genotypes: "0606" (lanes 1, 5, 6, 10–14, 16, 18–20, 22 and 24), "0202" (lanes 2–4) and "0608" (lanes 15, 17, 21 and 23). Lanes 7–9 are too faint to score, and were rerun. SSCP successfully detected small sequence differences; alleles 02 and 06 differ by one substitution, alleles 06 and 08 differ by a 3-bp deletion. In this example, homozygotes display two bands in the upper ssDNA banding system (one band per complementary strand), while heterozygotes display four (but see Discussion). Here, the lower double-stranded DNA banding system is uninformative. Running conditions were 15 W for 4 h at 4°C, 40 cm gel.

To identify and simultaneously isolate putative sequence- and/or size-variable alleles, isotope-labeled PCR products were subjected to SSCP following Sunnucks *et al*. [15]. Autoradiograph gel phenotypes consistent with expectations for a co-dominant diploid locus were identified (Figure 4), staple holes were used to align the film and dried gel, and positions of bands of interest were marked with a needle. Multiple alleles were excised (usually as ssDNA), soaked in TE, and incubated. Again, 1 μL of the supernatant was used for reamplification (PCR conditions follow step 1), then sequenced. For products generated using method A, one of the RAPD primers modified with a 10-bp 5' M13R(-20) tail (5'-TTCACACAGG-3') was used during reamplification, then sequenced using M13R(-20). Based on a multiple allele alignment, long (18- to 25-mer) locus-specific primers were designed in conserved regions flanking polymorphic sites. Amplified fragment lengths <350-bp were preferred, given that sensitivity of SSCP is less efficient for larger fragments [44].

### Selection of genotyping assays

We used a SSCP pre-screening step to assess suitability of new nuclear loci for yielding informative population-genetic data over the spatial scale of interest. Based on a subset of 20 individuals collected from diverse locations across the study site, copy number, ploidy and the minimum number of alleles at each locus were tentatively inferred from gel phenotypes. Loci with sufficient polymorphism and easily interpreted banding patterns (e.g. Figure 4) were targeted for large-scale SSCP screening of DNA sequence variation. Lower sensitivity assays such as RFLP or indel detection procedures were used for other loci.

### Genotyping procedures

Single-stranded conformation polymorphism coupled with targeted sequencing of alleles was used to genotype individuals on the basis of DNA sequence variation following Sunnucks *et al*. [15]. Radio-labeled PCR products were run on 6% non-denaturing acrylamide gels (40 cm) in a 4°C room. Run times and voltage were determined empirically (typically 5 to 11 h at 15 W, 13 mA). Samples with similar SSCP gel phenotypes were rerun side-by-side, and alleles from multiple representatives of each putative genotype per gel were excised, reamplified, and sequenced.

Restriction fragment length polymorphism assays were designed using NEBCUTTER V2.0 [45]. From the pre-screening step above, multiple alleles were selected for sequencing (common alleles were favored because they are more informative at the population-level). Restriction enzymes were chosen according to commercial availability, cost-effectiveness, and production of restriction site polymorphisms that could be easily resolved and for which *cis *and *trans *phase of multiple site heterozygotes could be unambiguously determined (see [3]). Composition of the 20-individual subset was rotated among loci to prevent potential biases on the marker characteristics [5]. Restriction digests were performed in 25 μL volumes containing: 2.5 μL recommended 10× Buffer (New England Biolabs, NEB), 2.5 μL spermidine trihydrochloride 40 mM (Sigma), 12.25 μL dH_2_O, 0.25 μL BSA 10 mg/mL (NEB), 2.5 u restriction enzyme, and 7 μL PCR product. Reactions were incubated (37°C for 12–16 h), and digestion products separated via electrophoresis following step 2.

Indels were assayed by electrophoresing [α^33^P]-dATP-labeled PCR products (amplified from genomic DNA) through 6% denaturing acrylamide gels (40 cm) for 2 h at 65 W, 40 mA, then viewed via autoradiography. Representatives of all observed alleles were sequenced to assess homology and determine the nature of the size variation.

### Sequence alignment, identity and function of nuclear gene regions

DNA sequencing was performed on an ABI 3730XL by Macrogen (Seoul, Korea). Sequences were edited with reference to chromatographs and aligned using ALIGNIR V2.0 (LI-COR). Areas of ambiguous alignment were omitted from the dataset. The identity of amplified gene regions was investigated using the nucleotide and protein BLAST search procedure [46]. Possible gene function was assessed by inspecting translated sequences for ORFs.

### Identification of population structure and assignment of individuals

A Bayesian clustering procedure using multi-locus genotype data, implemented in STRUCTURE V2.1 [47], was used to identify the number of populations (*K*), and assign individuals probabilistically to 'genetic' populations. Datasets for both species consisted of six nuclear loci. All runs employed the correlated allele frequency model and the admixture ancestry model. Estimated log likelihoods were obtained for *K *= 1 to *K *= 8, with five replicates of each *K*. A burn-in of 10^5 ^Markov chain Monte Carlo (MCMC) generations and run length of 10^6 ^MCMC generations was employed. All other parameters were default. The smallest value of *K *that captured the major structure in the data and identified geographically cohesive populations was considered correct. Unless otherwise stated, only individuals that were strongly assigned (*Q *≥ 0.90) to a single 'genetic' population were included in subsequent analyses given that admixed individuals cannot be unambiguously assigned. In cases where mtDNA data (not shown) identified a monophyletic clade nested within a single 'genetic' population, we separated those samples (and hence, erected an additional population) to reduce the chance of introducing Wahlund effect into analyses of nuclear loci. Full comparison of mtDNA and nuclear loci is outside the scope of this paper and will be presented elsewhere. Owing to relatively small sample sizes per population (Results), putative migrants were omitted from the datasets because they can potentially have a large impact on genetic diversity indices and obscure phylogeographic patterns.

### Levels of polymorphism and phenetic relationships among populations

Within-population and overall genetic variation of nuclear loci was assessed via allelic richness (*A*), calculated using a standardized sample size 14 diploid individuals in HP-RARE V1.0 [48]. Percentage of loci polymorphic (*P*) per population was calculated from the raw data. Phenetic relationships among populations were estimated via Neighbor-Joining (NJ) using Cavalli-Sforza & Edwards' [29]*D*_*c*_. Relative to other allele frequency-based genetic distances, *D*_*c *_is efficient in obtaining the correct tree topology under varying demographic scenarios, displays low sampling error [30], and is standardized with respect to drift [49]. To assess node support, we used PHYLIP V3.5c [50] to generate 1000 bootstrap distance matrices from which NJ trees were constructed, and then calculate the majority-rule (extended) consensus tree.

### Tests of recombination and selective neutrality

For loci assayed via SSCP coupled with targeted DNA sequencing, GENECONV V1.81 [51] was employed to evaluate evidence for recombination. Given that sensitivity and power of this analysis can be expected to increase with sampling effort, all observed alleles (including those present only in admixed and/or migrant individuals) were examined.

To test for departures from neutrality, Tajima's *D *[52] was calculated in DNASP V4.10.3 [53] for each population, and for all populations combined. While significant differences from the neutral expectation of zero can may indicate selection, they can also arise owing to population expansion, bottleneck, or heterogeneity of mutation rates among nucleotide sites. Since selection acts locally within the nuclear genome (whereas population demographic changes leave a common signature across multiple unlinked loci), we considered a significant departure from the neutral expectation at a given locus in all populations identified here as strong evidence of selection acting upon that locus. Conversely, when all loci displayed negative values of Tajima's *D *within a single population, we interpreted this as evidence for population growth (or decline). We acknowledge that population-specific selective sweeps affecting a single locus would be overlooked under this interpretation. Parameter sets and assessment of significance for tests of recombination and neutrality followed Garrick *et al*. [31].

### Phylogeographic analysis

Genealogical relationships among alleles at a locus were estimated using Templeton *et al*.'s [54] statistical parsimony, implemented in TCS V1.21 [55]. Alignment gaps were treated as a fifth character state, and contiguous gaps were coded as a single indel. Cladogram ambiguities were solved using predictions derived from coalescent theory, following Pfenninger & Posada's [56] frequency, topological and geographical criteria.

## Authors' contributions

RCG carried out the laboratory work, preformed data analyses and drafted the manuscript. PS identified the need to obtain three hierarchical levels of genetic information from the same locus, conceived the comprehensive marker development approach, and revised the manuscript. Both authors read and approved the final manuscript.
